# Power distance within student–teacher relationships: Let us talk about the rich pictures

**DOI:** 10.1111/medu.70098

**Published:** 2025-11-14

**Authors:** Gozie Offiah, Charlotte E. Rees

**Affiliations:** ^1^ Royal College of Surgeons in Ireland (RCSI) University of Medicine and Health Sciences Dublin Ireland; ^2^ Faculty of Medicine, Health and Life Science Swansea University Swansea UK; ^3^ Monash Centre for Scholarship in Health Education (MCSHE), Faculty of Medicine, Nursing and Health Sciences Monash University Melbourne Victoria Australia

## Abstract

.@gozie_offiah and Rees explore the possibility for multiple perspectives to be drawn from rich pictures studies to reflect the value of co‐construction between participants and investigators in our research practices.

In this issue, Ellawala and colleagues employ Hofstede's conception of power distance to explore student‐teacher power dynamics in medical education across Sri Lanka and the United Kingdom.^1^ Despite cultural differences, participants in both countries had similar perceptions of teacher authority, with power distance evident in both online and face‐to‐face learning environments. The authors' interpretations of rich pictures in their multimethod study are compelling and provoked us to consider further how visual and narrative data can be optimally integrated.^2^, ^3^ Therefore, we begin this commentary by building on the authors' rich picture interpretations, conducting our own ‘gallery walk’ of the drawings (i.e., interacting with and reflecting on the drawings),^2^, ^3^ and presenting additional researcher‐led analyses yielding alternative interpretations of power and triadic relationships, knowledge and gender. We do not have access to all the authors' data, so our interpretations are not meant to compete with those of the authors. Rather, we undertook this exercise to illustrate the multiplicity of plausible researcher‐led interpretations of rich pictures, and consequently encourage *Medical Education* readers to employ participant‐led interpretations of visual data, especially when addressing complex research questions, such as those related to power.

… we undertook this exercise to illustrate the multiplicity of plausible researcher‐led interpretations of rich pictures, and consequently encourage *Medical Education* readers to employ participant‐led interpretations of visual data …

Considering visual representations of power and relationships, while Ellawala et al focus only on dyadic student‐teacher relationships, we discerned alternative dyadic relationships (e.g., student–student, teacher–technology), as well as triadic relationships in the rich pictures. In Figures 1 and 6, the relationship was represented as dyadic (teacher–technology), suggesting that students were depicted as technology (and therefore absent) in online learning, potentially reflecting their disempowerment. Indeed, the rich pictures also depict a multiplicity of triadic relationships, underscoring the complexity of power dynamics. For example, in Figure 7, we observed triadic student–teacher–technology relationships. We also observed the student‐teacher–patient relationship (Figure 3), not commented on by the authors, with the patient positioned at the periphery in this triadic relationship, again illustrating patient disempowerment. Finally, the first picture in Figure 1 shows student–teacher–peer relationships in face‐to‐face learning, depicted by double‐headed arrows between teacher–student and student–student, potentially illustrating more shared power and student empowerment.

… the rich pictures depict a multiplicity of triadic relationships, underscoring the complexity of power dynamics.

While the study authors do not specifically talk about the relationship between power and knowledge in their interpretations, they do highlight how teachers were often depicted as larger than students (Figures 1–4 and 6), which may suggest participants' perceptions of teachers as more powerful and knowledgeable. In relation to one figure in particular (Figure 4), the authors highlight how physical objects (i.e., the whiteboard) and teaching space arrangements (i.e., the teacher talking next to the whiteboard, facing the students in rows) similarly portray teacher power. Building on this interpretation, we were especially struck by the participant's representation of the teacher's disproportionately large head, which could symbolize cognitive dominance, reinforcing the idea of knowledge transmission from teacher to student in traditional classroom settings. Some drawings also seemed to us to reveal attempts to humanize the teacher, reduce power distance and foster inclusivity. For example, the teacher positioning themselves among students (Figure 5), using open body language, and avoiding physical symbols of power.

… we were especially struck by the participant's representation of the teacher's disproportionately large head, which could symbolize cognitive dominance …

The rich pictures also offer interesting representations of power in relation to gender, as when Ellawala et al interpret a picture of a teacher with a hands‐on‐hips posture as depicting power (Figure 3). We considered that such a posture could convey a range of meanings, including teacher confidence, authoritativeness, bossiness, discontent and so on. In doing so, however, we were struck by the limited discussion of the gendered representation of this teacher, drawn with long curly hair and a triangular dress (as female), compared with other gender‐neutral stick figures. Would a male teacher adopting the same posture be interpreted as confident rather than enacting power? Furthermore, Figure 2 presents a gender‐neutral teacher (a stick figure with short curly hair and elongated arms) encircling their student cohort. The authors describe the individual as both ‘parental’ and ‘nurturing’. At first glance, then, one possible interpretation is that this teacher is female, with her arms enfolding the students in a caring hug. However, if we thought this teacher stick figure represented a male, then the arms enfolding the students might be considered to be protective and/or controlling. Again, that is not to suggest that one interpretation is right or wrong; rather, the difference draws attention to how gendered interpretations could reflect deeply ingrained societal biases and stereotypes (e.g., nurturing = female; protective = male).^4^

The rich pictures also offer interesting representations of power in relation to gender, as when Ellawala et al interpret a picture of a teacher with a hands‐on‐hips posture as depicting power …

Through these alternative interpretations, we hope to have illustrated how rich pictures can have multiple meanings to different people, and how our own researcher backgrounds, understandings, experiences and biases will influence interpretations of visual stimuli.^5^ For that reason, Cristancho and Helmich question the appropriateness of stand‐alone researcher‐led analyses of rich pictures, arguing that conversation with participants *about* their drawings is a crucial step in the use of rich pictures.^2^, ^3^ Similarly, Conte and Davidson highlight how visual metaphors not only depict complex systems but also serve as tools for participatory sense‐making.^6^ So, while the authors use rich pictures to illustrate relational power dynamics (allowing for the expression of complex, often unspoken dynamics that textual data alone might miss^6^), we would also like to know whether participant‐researcher joint sense‐making of the pictures might have added more diverse perspectives. Interpretation is inevitable in studies of this type, so we are not arguing for a right or wrong answer. Still, complex phenomena (such as power in student‐teacher and other relationships and its relation to knowledge and gender) deserve rich, nuanced interpretations that can best be achieved through co‐construction involving researchers and participants. That is, rather than participants drawing and talking (separately) as a method of data collection and researcher‐led interpretations of rich pictures, participants should be encouraged to talk about their drawings and co‐analyse them with researchers. Thus, we urge medical education researchers to consider participatory visual methodologies from a critical inquiry (rather than interpretivist) perspective, to facilitate egalitarian researcher–participant relationships and to address complex research questions about power.^7^

Complex phenomena (such as power in student‐teacher and other relationships and its relation to knowledge and gender) deserve rich, nuanced interpretations that can best be achieved through co‐construction involving researchers and participants.

## AUTHOR CONTRIBUTIONS

Both authors, Gozie Offiah and Charlotte Rees, contributed equally to the conception and design of the commentary, as well as the drafting and reviewing of the intellectual content and approved the commentary in its final form.

## Data Availability

The data that support the findings of this study are openly available in University of Dundee at https://discovery.dundee.ac.uk/en/studentTheses/women‐in‐surgery.
